# Characterization of diabetes following pancreatic surgery in patients with congenital hyperinsulinism

**DOI:** 10.1186/s13023-018-0970-8

**Published:** 2018-12-22

**Authors:** Alena Welters, Thomas Meissner, Jürgen Grulich-Henn, Elke Fröhlich-Reiterer, Katharina Warncke, Klaus Mohnike, Oliver Blankenstein, Ulrike Menzel, Nicolin Datz, Esther Bollow, Reinhard W. Holl

**Affiliations:** 1 Department of General Paediatrics, Neonatology and Paediatric Cardiology, University Children’s Hospital Düsseldorf, Moorenstrasse 5, 40225 Düsseldorf, Germany; 2 Centre for Childhood and Adolescent Medicine (General Paediatrics), University Hospital Heidelberg, Heidelberg, Germany; 3 Department of Paediatrics, Medical University of Graz, Graz, Austria; 4 Department of Paediatrics, Klinikum rechts der Isar, Technische Universität München, Munich, Germany; 5 Department of Paediatrics, Otto von Guericke University Magdeburg, Magdeburg, Germany; 6 Centre for Chronic Sick Children, Institute for Experimental Paediatric Endocrinology, Charité - Universitätsmedizin Berlin, Berlin, Germany; 7 Department of Paediatric Endocrinology, AKK Altonaer Kinderkrankenhaus, Hamburg, Germany; 8 Diabetes Centre for Children and Adolescents, Children’s Hospital AUF DER BULT, Hannover, Germany; 9 Institute of Epidemiology and Medical Biometry, ZIBMT, University of Ulm, Ulm, Germany; 10 German Centre for Diabetes Research (DZD), Munich, Neuherberg Germany

**Keywords:** Congenital hyperinsulinism, Diabetes, Pancreatectomy, DPV initiative

## Abstract

**Background:**

Congenital hyperinsulinism (CHI) is the most common cause of persistent hypoglycaemia in infancy that leads to unfavourable neurological outcome if not treated adequately. In patients with severe diffuse CHI it remains under discussion whether pancreatic surgery should be performed or intensive medical treatment with the acceptance of recurrent episodes of mild hypoglycaemia is justified. Near-total pancreatectomy is associated with high rates of insulin-dependent diabetes mellitus and exocrine pancreatic insufficiency. Little is known about the management and long-term glycaemic control of CHI patients with diabetes after pancreatic surgery. We searched the German/Austrian DPV database and compared the course of 42 CHI patients with diabetes to that of patients with type 1 diabetes mellitus (T1DM). Study groups were compared at diabetes onset and after a follow-up period of 6.1 [3.3–9.7] (median [interquartile range]) years.

**Results:**

The majority of CHI patients with diabetes were treated with insulin (85.2% [70.9–99.5] at diabetes onset, and 90.5% [81.2–99.7] at follow-up). However, compared to patients with T1DM, significantly more patients in the CHI group with diabetes were treated with conventional insulin therapy (47.8% vs. 24.4%, *p* = 0.03 at diabetes onset, and 21.1% vs. 6.4% at follow-up, *p* = 0.003), and only a small number of CHI patients were treated with insulin pumps. Daily insulin dose was significantly lower in CHI patients with diabetes than in patients with T1DM, both at diabetes onset (0.3 [0.2–0.5] vs. 0.6 IE/kg/d [0.4–0.8], *p* = 0.003) and follow-up (0.8 [0.4–1.0] vs. 0.9 [0.7–1.0] IE/kg/d, *p* = 0.02), while daily carbohydrate intake was comparable in both groups. Within the first treatment year, HbA1c levels were significantly lower in CHI patients with diabetes (6.2% [5.5–7.9] vs. 7.2% [6.5–8.2], *p* = 0.003), but increased to a level comparable to that of T1DM patients at follow-up. Interestingly, in CHI patients, the risk of severe hypoglycaemia tends to be higher only at diabetes onset (14.8% vs. 5.8%, *p* = 0.1).

**Conclusions:**

In surgically treated CHI patients insulin treatment needs to be intensified in order to achieve good glycaemic control. Our data furthermore emphasize the need for improved medical treatment options for patients with diazoxide- and/or octreotide-unresponsive CHI.

## Background

Congenital hyperinsulinism (CHI) is a heterogeneous metabolic disorder that is characterized by the unregulated release of insulin from pancreatic beta cells leading to recurrent episodes of hypoglycaemia [1]. CHI is a rare disease, affecting approximately 1 in 50.000 newborns in Europe and the United States, but the most common cause of persistent hypoglycaemia in infancy [2]. Rapid diagnosis and adequate therapy that aims at maintaining blood glucose concentrations within a physiological range are crucial to prevent hypoglycaemic brain damage and to achieve good neurodevelopmental outcomes [3–8]. Management of CHI involves nutritional, medical and surgical intervention, depending on the underlying histologic and genetic subtype of CHI [1]. Management of diffuse CHI (DCHI) that accounts for most CHI cases still remains a major challenge. Most patients require a combination of different medications, including off-label use of drugs like somatostatin analogues or sirolimus, and additional nutritional therapy to achieve sufficient glycaemic control [9–14]. Importantly, in many medically treated CHI patients disease severity reduces over time [15, 16]. Still, there is a persisting risk of hypoglycaemia and subsequent neurodevelopmental impairment in medically and nutritionally treated DCHI. In those patients with severe medically-unresponsive DCHI near-total pancreatectomy, i.e. resection of approximately 95–98% of pancreatic tissue may be required [17, 18]. The outcome of patients with DCHI treated with near-total pancreatectomy is variable and often unsatisfactory. High rates of persisting hypoglycaemia (up to 60%), hyperglycaemia (almost 100% at 11 years post-surgery) and exocrine pancreatic insufficiency (almost 50%) have been reported in patients with DCHI following pancreatectomy [19–23]. Even though all CHI patients treated by near-total pancreatectomy eventually develop insulin-dependent diabetes mellitus, very little is known about the characteristics of this specific diabetes type, particularly the intensity of diabetes management, the associated risk of hypoglycaemia and long-term glycaemic control.

We chose a multi-centre approach and searched the German/Austrian Diabetes Patienten Verlaufsdokumentation (DPV) database to compare the course of 42 CHI patients with diabetes to that of age-matched patients with type 1 diabetes mellitus (T1DM). Data were compared at diabetes onset and after a median follow-up period of 6.1 [3.3–9.7] years (median [interquartile range]). Here we provide objective information on treatment modality (conventional insulin therapy, intensified insulin therapy, insulin pump), insulin dose and daily carbohydrate intake, glycaemic control and the risk of hypoglycaemia in CHI patients following pancreatic surgery. Our data emphasize the need to reconsider the management and treatment goals in this particular group of diabetes patients, and strengthen the need for alternative treatment options for patients with DCHI.

## Methods

### DPV registry

Data were extracted from the DPV registry, a nationwide prospective multicentre initiative that records demographic and clinical data of children and adults with any type of diabetes. More than 400 centres in Germany, Austria, Switzerland and Luxembourg participate in the DPV initiative. Each centre transmits its data biannually in an anonymous form to the University of Ulm, Germany for central data acquisition and analysis. Data are screened for inconsistency and, if applicable, reported back to centres for re-confirmation or correction. Until September 2016 471.247 patients with diabetes were registered in the electronic computer based documentation software DPV. The DPV initiative and the analysis of anonymized data related to quality of care were approved by the ethics committee of the University of Ulm.

For the present analysis we included all subjects with T1DM or congenital hyperinsulinism and pancreatic surgery aged younger than 26 years. For each patient, data from the first treatment year (duration of diabetes less than 1 year) and from the most recent treatment year were extracted and analysed.

The final study sample encompassed 54.747 and 65.982 patients with T1DM, and 27 and 42 patients with CHI and diabetes at diabetes onset and follow-up, respectively.

### Data analysis

Diabetes management was categorized as insulin therapy or therapy with oral antidiabetic drugs (OADs). Insulin therapy was further subclassified as (1) conventional insulin therapy (CT), if 1–3 times of injections per day were documented, or (2) intensified insulin therapy (ICT), if 4–8 times of injections per day were documented, or (3) continuous subcutaneous insulin infusion (CSII). Insulin requirements are expressed as total daily insulin dose (IE/d) and daily insulin dose per kilogram (kg) body weight (IE/kg/d). Carbohydrate intake was calculated in carbohydrate units (CU, one unit equals about 12 g carbohydrates), and expressed as total daily carbohydrate intake (CU/d) and daily carbohydrate intake per kg body weight (CU/kg/d). Glycaemic control was determined by glycated haemoglobin A1c (HbA1c) level. HbA1c level from different centres were mathematically standardized to the reference range of the Diabetes Control and Complication Trial (DCCT) (4.05–6.05%). Severe hypoglycaemia was defined according to the ISPAD guidelines, i.e. an episode of hypoglycaemia associated with severe cognitive impairment requiring external assistance by another person [24]. Body mass index (BMI) was calculated as weight in kg divided by square of the height in meters (kg/m^2^). C-Peptide secretion in μg/L was categorized as either < 1 μg/L (negative) or > 1 μg/L (positive).

### Statistics

Descriptive statistics are given as median [Q1-Q3] or as percentages. Differences between individuals with T1DM and subjects with CHI and diabetes were analysed using Kruskal-Wallis test for continuous variables and χ^2^-test for dichotomous variables. False discovery rate was used to correct for multiple comparisons. Comparisons between the first year after manifestation and the most recent treatment year were calculated by using t-test for continuous variables, and McNemar test for dichotomous variables. A two-sided *p*-value < 0.05 was considered significant. SAS version 9.4 software (SAS Institute, Cary, NC, USA) was used for statistical analysis.

## Results

### Patient characteristics

**Fig. 1:**
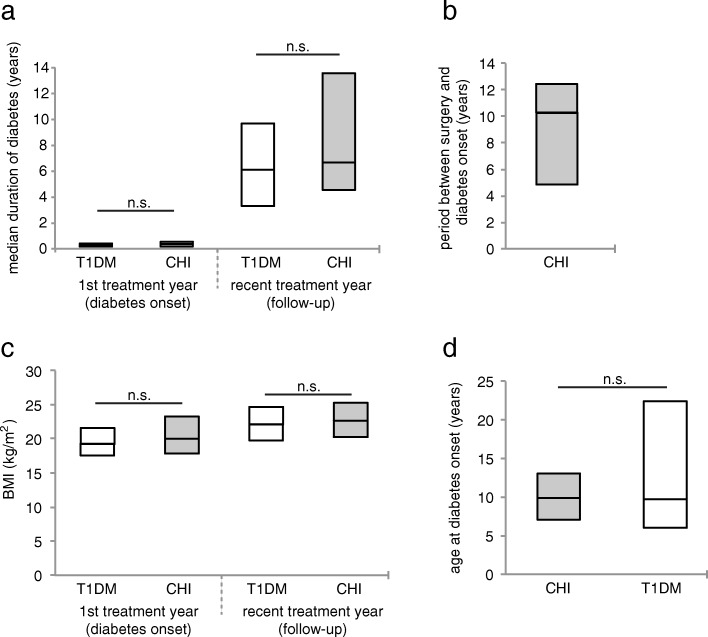
Selected characteristics of CHI patients with diabetes compared to patients with T1DM. **a** Median duration of diabetes (years); *n* = 27 (diabetes onset) and 42 (follow-up) CHI patients with diabetes, and *n* = 54,747 (diabetes onset) and 65,982 (follow-up) patients with T1DM; **b** Median period between pancreatic surgery and diabetes onset in CHI patients with diabetes; *n* = 22; **c** BMI (kg/m^2^); *n* = 19 (diabetes onset) and 37 (follow-up) CHI patients with diabetes, and *n* = 33,326 (diabetes onset) and 58,388 (follow-up) patients with T1DM **d** Age at diabetes onset; *n* = 27 CHI patients with diabetes, and *n* = 54,774 patients with T1DM. Significance determined by *p* < 0.05 using Wilcoxon test

Fourty-five patients with CHI and diabetes following pancreatic surgery are currently registered in the German/Austrian DPV database. Data on diabetes management, insulin requirements, carbohydrate intake, glycaemic control (HbA1c level), and C-peptide secretion were available for up to 27 of these patients from the first year after manifestation (hereafter specified as “diabetes onset” data), and for up to 42 of these patients from the most recent treatment year (hereafter specified as “follow-up” data). Patients with T1DM served as control and were directly compared to the CHI patients with diabetes (Table 1). Data at diabetes onset were obtained after a median duration of diabetes of 0.4 (0.2–0.5; *p* = 0.3) years in the CHI group versus *(*vs.*)* 0.3 (0.2–0.5) years in the T1DM group. At follow-up, median duration of diabetes was 6.7 (4.6–13.6; *p* = 0.12) years in the CHI group vs. 6.1 (3.3–9.7) years in the T1DM group (Fig. 1a). In CHI patients median period between pancreatic surgery and diabetes manifestation was 10.3 [4.9–12.5] years (Fig. 1b).

**Table 1:** Study sample

	1st treatment year (diabetes onset)	recent-treatment year (follow-up)
n patients (T1DM and CHI)	54,774	66,024
n patients with CHI	27	42
age (years)	10 [6.3–13.3]	16.3 [12.9–17.9]
male (%)	53.8	52.4
BMI (kg/m^2^)	19.2 [17.5–21.6]	22.1 [19.7–24.7]

There was no significant difference in BMI (19.2 [17.5–21.6] kg/m^2^ in T1DM patients vs. 20.0 [17.8–23.3] kg/m^2^ in CHI patients, and 22.1 [19.7–24.7] kg/m^2^ in T1DM patients vs. 22.6 [20.3–25.3] kg/m^2^ in CHI patients at diabetes onset and follow-up, respectively; *p* = 0.6 and *p* = 0.9) and age at diabetes onset (9.8 [7.0–13.0] years in the CHI group, and 9.7 [6.0–13.0] in the T1DM group, *p* = 0.7) (Fig. 1c,d).

### A large number of patients with CHI and diabetes are treated with conventional insulin therapy, and only a small number with insulin pumps

**Fig. 2:**
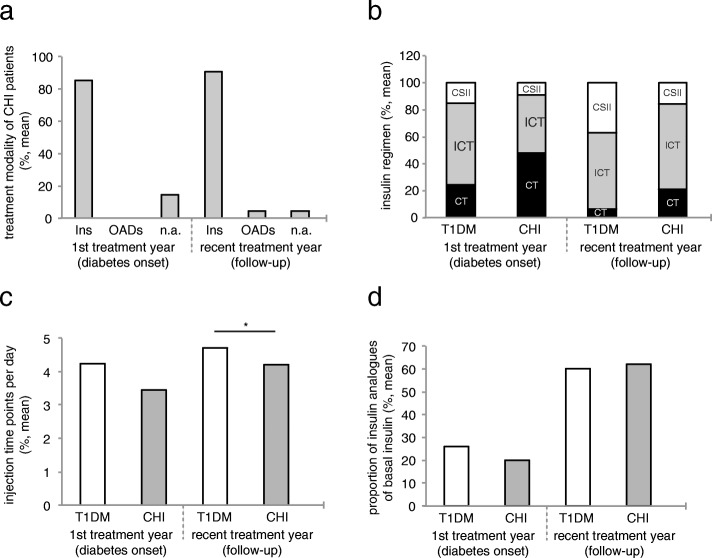
Management of diabetes in CHI patients with diabetes compared to patients with T1DM. **a** Proportion of CHI patients with diabetes treated with insulin or oral antidiabetic drugs; *n* = 27 (diabetes onset) and 42 (follow-up) CHI patients with diabetes. **b** Insulin regimen in CHI patients with diabetes compared to patients with T1DM; *n* = 23 (diabetes onset) and 38 (follow-up) CHI patients with diabetes, and *n* = 51,704 (diabetes onset) and 62,829 (follow-up) patients with T1DM. **c** Number of injection times per day in CHI patients with diabetes compared to patients with T1DM; *n* = 20 (diabetes onset) and 29 (follow-up) CHI patients with diabetes, and *n* = 43,378 (diabetes onset) and 39,546 (follow-up) patients with T1DM. **d** Proportion of insulin analogues as basal insulin supplementation; *n* = 20 (diabetes onset) and 29 (follow-up) CHI patients with diabetes, and *n* = 43,378 (diabetes onset) and 39,546 (follow-up) patients with T1DM. All values are means. *P < 0.05. Significance determined by *p* < 0.05 using χ^2^-test

The vast majority of CHI patients with diabetes were treated with insulin (85.2% at diabetes onset, and 90.5% at follow-up). At follow-up a small proportion of CHI patients with diabetes were treated with OADs (4.8%), of which 2,4% were treated with metformin (Fig. 2a). We further compared insulin regimen between CHI patients with diabetes and T1DM patients at diabetes onset and at follow-up, i.e. the proportion of patients treated with CT, ICT and CSII (Fig. 2b). Both, within the first treatment year, but also at follow-up, significantly more patients in the CHI group with diabetes were treated with CT (47,8% vs. 24.4% patients with T1DM, *p* = 0.03; and 21.1% vs. 6.4% patients with T1DM at follow-up, *p* = 0.003), and only a small number of CHI patients were treated with CSII (8.7% vs. 15.2% patients with T1DM, *p* = 0.48; and 15.8% [3.6–27.9] vs. 36.7% patients with T1DM at follow-up, *p* = 0.03). Within the first treatment year, the majority of T1DM patients were treated with ICT (60.3%) whereas the majority of CHI patients with diabetes were treated with CT (47.8%). However, at follow-up, standard treatment for both, CHI patients and T1DM patients was ICT (63.2% vs. 56.9% patients with T1DM, *p* = 0.56). We also analysed the number of injection times per day in CHI patients with diabetes and T1DM patients (Fig. 2c): there was no significant difference at diabetes onset, whereas at follow-up, T1DM patients had significantly more injection time points per day than CHI patients with diabetes (4.3 vs. 3.5 within the first treatment year, *p* = 0.19 and 4.7 vs. 4.2 at follow-up, *p* = 0.03). Within the first treatment year there was furthermore a tendency towards a lower proportion of insulin analogues used as basal insulin supplementation in CHI patients with diabetes (20% vs. 26% in patients with T1DM, *p* = 0.8). However, at follow-up the use of insulin analogues had increased 3-fold and was similar in both groups (62.1% vs. 60.2% in patients with T1DM, *p* = 0.77).

### Within the first year of antidiabetic treatment, the risk of severe hypoglycaemia is high in CHI patients with diabetes

**Fig. 3:**
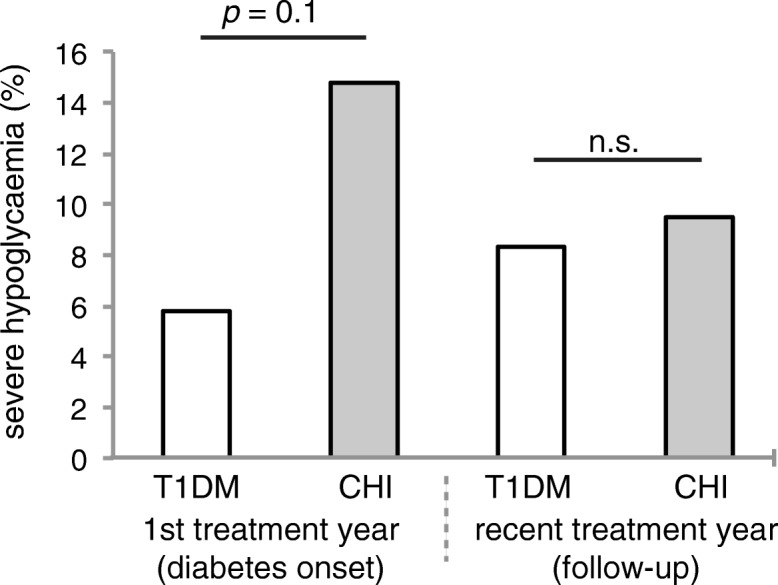
Frequency of severe hypoglycaemia in CHI patients with diabetes compared to patients with T1DM. Severe hypoglycaemia; *n* = 27 (diabetes onset) and 42 (follow-up) CHI patients with diabetes, and *n* = 54,706 (diabetes onset) and 65,927 (follow-up) patients with T1DM. All values are means. Significance determined by *p* < 0.05 using χ^2^-test (and McNemar test)

The risk of severe hypoglycaemia tends to be higher in CHI patients with diabetes than in patients with T1DM, particularly within the first year of treatment (14.8% vs. 5.8% patients with T1DM, *p* = 0.11) (Fig. 3). However, whereas severe hypoglycaemia was relatively frequent within the first year of treatment in CHI patients, the risk decreased to a proportion comparable to that of T1DM patients at follow-up (9.5% vs. 8.3% patients with T1DM, *p* = 0.8).

### Within the first treatment year, glycaemic control is relatively good in CHI patients with diabetes, but significantly deteriorates as diabetes progresses

While patients with T1DM typically had an increased HbA1c level at diabetes onset (7.2% [6.5–8.2]), this was not always the case in patients with CHI (6.2% [5.5–7.9], *p* = 0.003.

**Fig. 4:**
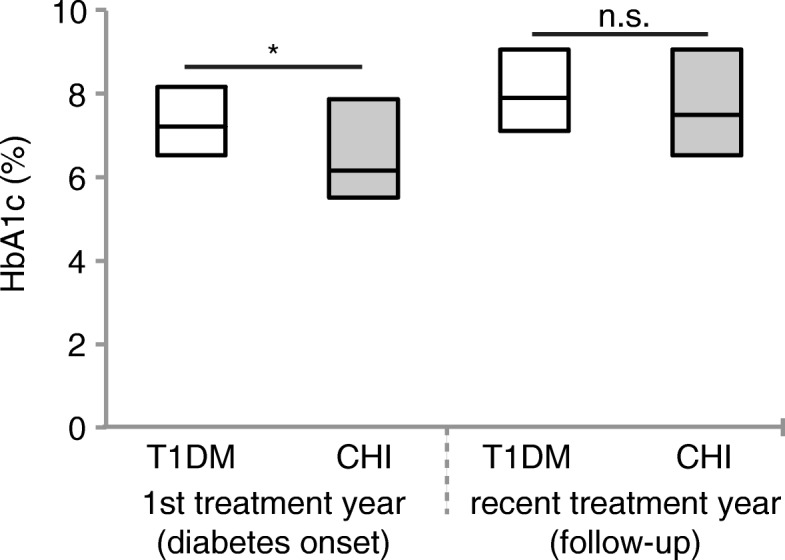
Glycaemic control in CHI patients with diabetes compared to patients with T1DM. HbA1c level; *n* = 25 (diabetes onset) and 42 (follow-up) CHI patients with diabetes, and *n* = 52,825 (diabetes onset) and 63,368 (follow-up) patients with T1DM. All values are median ± lower and upper quartile. *P < 0.05. Significance determined by *p* < 0.05 using Wilcoxon test (and t-test)

However, as diabetes progressed, glycaemic control significantly worsened in both groups, and at follow-up CHI patients had reached a similar level of control as patients with T1DM (HbA1c 7.5% [6.5–9.1] vs. 7.9% [7.1–9.1], *p* = 0.12) (Fig. 4).

### In CHI patients with diabetes, C-peptide secretion progressively declines as diabetes progresses

**Fig. 5:**
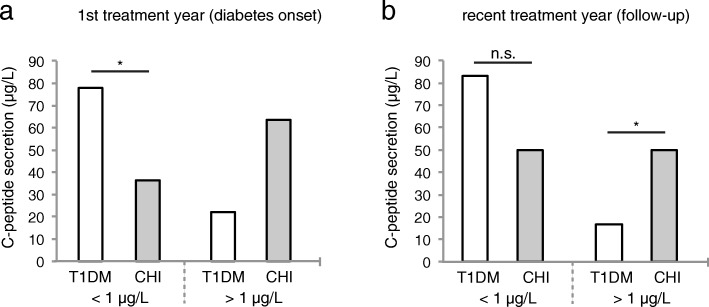
Residual beta cell function at diabetes onset (**a**) and at follow-up (**b**) in CHI patients with diabetes compared to patients with T1DM. C-peptide secretion; *n* = 11 (diabetes onset) and 8 (follow-up) CHI patients with diabetes, and *n* = 13,746 (diabetes onset) and 3497 (follow-up) patients with T1DM. All values are means. Significance determined by *p* < 0.05 using χ^2^-test

For the evaluation of C-peptide secretion as a marker of residual beta cell function, patients were assigned to one of two categories: (I) C-peptide secretion < 1 μg/L (negative), (II) C-peptide secretion > 1 μg/L (positive) (Fig. 5). Within the first treatment year, the majority of T1DM patients fell into the first category (77.9%), whereas the majority of CHI patients with diabetes fell into category (II) (63.6%) (Fig. 5a). As diabetes progressed, C-peptide secretion declined in both, T1DM patients and CHI patients with diabetes. However, compared to patients with T1DM, at follow-up significantly more patients in the CHI group could still be assigned to the second category (50% vs. 16.8%, *p* = 0.04) (Fig. 5b).

### Daily insulin dose is relatively low in CHI patients with diabetes. Daily carbohydrate intake is comparable to that of type 1 diabetic patients

**Fig. 6:**
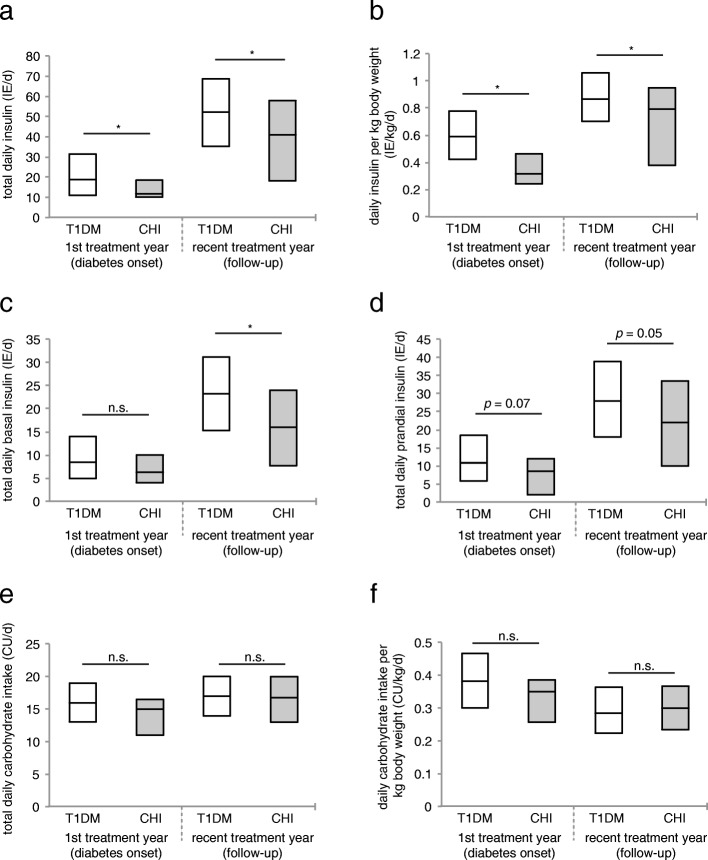
Insulin requirement and carbohydrate intake in CHI patients with diabetes compared to patients with T1DM. **a** Total daily insulin dose (IE/d); *n* = 23 (diabetes onset) and 38 (follow-up) CHI patients with diabetes, and *n* = 51,704 (diabetes onset) and 62,829 (follow-up) patients with T1DM. **b** Daily insulin dose per kg body weight (IE/kg/d); *n* = 18 (diabetes onset) and 35 (follow-up) CHI patients with diabetes, and *n* = 32,304 (diabetes onset) and 57,206 (follow-up) patients with T1DM. **c** Total daily basal insulin dose (IE/d); *n* = 22 (diabetes onset) and 35 (follow-up) CHI patients with diabetes, and *n* = 51,145 (diabetes onset) and 62,440 (follow-up) patients with T1DM. **d** Total daily prandial insulin dose (IE/d); *n* = 21 (diabetes onset) and 37 (follow-up) CHI patients with diabetes, and *n* = 50,454 (diabetes onset) and 62,246 (follow-up) patients with T1DM. **e** Total daily carbohydrate intake (CU/d); *n* = 18 (diabetes onset) and 34 (follow-up) CHI patients with diabetes, and *n* = 45,494 (diabetes onset) and 58,655 (follow-up) patients with T1DM. **f** Daily carbohydrate intake per kg body weight (CU/kg/d); *n* = 16 (diabetes onset) and 31 (follow-up) CHI patients with diabetes, and *n* = 28,202 (diabetes onset) and *n* = 53,380 (follow-up) patients with T1DM. All values are median ± lower and upper quartile. *P < 0.05. Significance determined by *p* < 0.05 using Wilcoxon test

Compared to patients with T1DM, total daily insulin dose and insulin dose per kg body weight were significantly lower in CHI patients with diabetes, both within the first treatment year and at follow-up (Fig. 6a and b): daily insulin dose was 11.8 IE [10.1–18.5] vs. 18.8 IE [11–31.5] in patients with T1DM, *p* = 0.03 at diabetes onset, and 41 IE [18.2–58] vs. 52.3 IE [35.3–68.7] in patients with T1DM, *p* = 0.02 at follow-up; insulin dose per kg body weight was 0.3 IE [0.2–0.5] vs. 0.6 IE [0.43–0.78] in patients with T1DM, *p* = 0.003 at diabetes onset, and 0.8 IE [0.4–1.0] vs. 0.9 IE [0.7–1.1] in patients with T1DM, *p* = 0.02 at follow-up.

Daily basal insulin dose tended to be lower in CHI patients with diabetes within the first treatment year (6.3 IE [4–10] vs. 8.4 IE [5–14] in patients with T1DM, *p* = 0.12), a trend that reached significance as diabetes progressed (16 IE [7.7–24] vs. 23.2 IE [15.2–31.1] in patients with T1DM, *p* = 0.02 at follow-up) (Fig. 6c). Surprisingly, related to total daily insulin, basal insulin requirements were relatively high in patients with CHI within the first treatment year (53.2% of total daily insulin vs. 44.7% in patients with T1DM), but markedly declined over time (39% vs. 44.4% in patients with T1DM at follow-up).

Daily prandial insulin dose were lower in CHI patients with diabetes both, at diabetes onset (8.5 IE [2–12] vs. 10.8 IE [5.9–18.5] in patients with T1DM, *p* = 0.07) and at follow-up (22 IE [10–33.5] vs. 28 IE [18–38.8] in patients with T1DM, *p* = 0.05) (Fig. 6d).

Interestingly, there was no significant difference in total daily carbohydrate intake and daily carbohydrate intake per kg body weight between CHI patients with diabetes and those with T1DM. In fact, in CHI patients, reported daily carbohydrate intake tended to be slightly lower compared to patients with T1DM (Fig. 6e and f): reported total daily carbohydrate intake was 15CU [11–16.5] vs. 16CU [13–19] in patients with T1DM, *p* = 0.19 at diabetes onset, and 16.8CU [13–20] vs. 17CU [14–20] in patients with T1DM, *p* = 0.57 at follow-up; carbohydrate intake per kg body weight was 0.4CU [0.3–0.4] vs. 0.4CU [0.3–0.5] in patients with T1DM, *p* = 0.13 at diabetes onset, and 0.3CU [0.2–0.4] vs. 0.3CU [0.2–0.4] in patients with T1DM, *p* = 0.57 at follow-up.

## Discussion

Patients with severe diffuse CHI typically require prolonged nutritional and medical treatment to avoid episodes of severe symptomatic hypoglycaemia and to maintain blood glucose concentrations within a range regarded as safe with respect to brain damage [9].

The development of new drug formulations, e.g. synthetic somatostatin analogues with prolonged half-life, facilitated medical treatment of DCHI to some extent [9–12, 25–27]. More recently, the efficacy of the mammalian target of rapamycin (mTOR) inhibitor sirolimus has been investigated in critically ill CHI patients unresponsive to diazoxide and octreotide [13]. Successful outcomes have been achieved in some patients treated with sirolimus, including a neonate with severe HH in Beckwith-Wiedemann Syndrome [28–32]. However, others have published on limited therapeutic success at the expense of serious side effects, and therefore extreme cautious use of sirolimus has been advised in children with CHI [14, 33, 34]. In the past, many medically-unresponsive DCHI patients underwent extensive pancreatectomy in an ultimate attempt to prevent severe hypoglycaemia [18, 35]. Still, near-total pancreatectomy (typically a 95% resection) remains the last resort to prevent hypoglycaemic brain damage in medically unresponsive severe DCHI [1, 35]. However, it has widely been proven in previous studies that surgical intervention is never curative in children with DCHI [7, 19–21, 23, 36, 37]. In fact, the long-term results of surgical intervention in children with DCHI are very unsatisfactory. Several groups revealed high incidence rates of diabetes mellitus after extensive pancreatectomy (> 85%) [19–23]. Long-term follow up data prove that in CHI patients the incidence of insulin-dependent diabetes mellitus is almost 100% 10–15 years after near-total pancreatectomy [19, 20]. Given that almost all DCHI patients treated with near-total pancreatectomy eventually develop diabetes mellitus, surprisingly little is known about their management and long-term glycaemic control. Recommendations how intense these patients can or should be treated do not exist. Treatment regimen of these patients therefore mostly depends on single-centre experiences.

Our data reveal that most CHI patients with diabetes appear to be treated less intense than T1D patients, as significantly more CHI patients with diabetes are treated with conventional insulin therapy, both at diabetes onset and at follow-up. This is also expressed by the overall number of injection times per day, which tends to be lower in CHI patients, particularly at follow-up, and by the less common use of basal insulin analogues within the first treatment year. Furthermore, only a very small number of CHI patients with diabetes are treated with insulin pumps (Fig. 2). This might be ascribed to the large proportion of CHI patients with residual beta cell function at diabetes onset (Fig. 5), and/or reflect the physician’s attempt to minimize the risk of hypoglycaemia. In fact, severe hypoglycaemia is more frequent in CHI patients with diabetes than in patients with T1DM, probably due to unregulated release of insulin from the remaining, yet malfunctioning beta cells. Impaired counterregulatory response to hypoglycaemia owing to glucagon deficiency, and enhanced peripheral insulin sensitivity that has been shown in adults with pancreatogenic diabetes (i.e. diabetes secondary to diseases of the exocrine pancreas or pancreatectomy) further increase the risk of hypoglycaemia after pancreatic surgery [38–40]. Interestingly, our data reveal that the risk of severe hypoglycaemia tends to be higher only within the first treatment year, while it decreases to a rate comparable to that of T1DM patients as diabetes progresses (Fig. 3). Therefore, in CHI patients with diabetes, a more intensive approach in the course of diabetes seems to be feasible, particularly with regard to the level of glycaemic control achieved in these patients at follow-up: at diabetes onset, some CHI patients with diabetes still had normal HbA1c level, while at follow-up HbA1c had increased significantly. However, it has to be taken into consideration that in CHI patients with early diabetes postprandial hyperglycaemia typically alternate with recurrent episodes of hypoglycaemia and therefore HbA1c initially remains low. As a measure of residual beta cell function, we furthermore evaluated random C-peptide secretion. Both, at diabetes onset and at follow-up, endogenous insulin release is higher in CHI patients with diabetes than in patients with T1DM, apparently due to the remaining beta cell mass (Fig. 5). Consistently, insulin requirements are lower in CHI patients with diabetes compared to patients with T1DM, particularly as diabetes progresses (Fig. 6 a,b). Residual secretory capacity and enhanced peripheral insulin sensitivity probably account for low insulin requirements of CHI patients with diabetes [40]. However, a persisting tendency towards hypoglycaemia and fear of hypoglycaemia may also have an impact on basal and prandial insulin dose.

## Conclusion

This multicentre approach provides objective information on the management of CHI patients with diabetes following pancreatic surgery. Of note, the diabetes registry includes only diabetes-related data. Limited data were available about the treatment prior to diabetes, the extent of pancreatectomy (i.e. partial vs. subtotal vs. near total pancreatectomy) and about the underlying genetics of CHI. As extensive pancreatic resection appears to be a prerequisite for the development of diabetes, we presume that CHI patients in our cohort were treated by subtotal or near-total pancreatectomy [7, 35].

Our data indicate, that CHI patients with diabetes often require an intensive insulin therapy comparable to that of type 1 diabetic patients, particularly when diabetes progresses and residual beta cell function further declines. At this time, a more rigorous insulin regimen is necessary to improve the long-term metabolic outcome of diabetic CHI patients, particularly with respect to diabetic long-term complications. This means that in those infants with severe diffuse CHI intensive medical treatment including off-label use of drugs, nutritional therapy and acceptance of recurrent episodes of mild hypoglycaemia has to be weighed against the long-term risks and side effects of surgical management. In view of this dilemma, i.e. poor outcome and/or a high burden for patients and family members with both approaches, there is an urgent need for alternative medical treatment options for patients with CHI.

## Abbreviations

BMI: Body mass index
CHI: Congenital hyperinsulinism
CSII: Continuous subcutaneous insulin infusion
CT: Conventional insulin therapy
CU: Carbohydrate unit
DCCT: Diabetes Control and Complication Trial
DCHI: Diffuse congenital hyperinsulinism
DPV: Diabetes Patienten Verlaufsdokumentation
FCHI: Focal congenital hyperinsulinism
HbA1c: Glycated haemoglobin A1c
ICT: Intensified insulin therapy
IE: Internationale Einheit
Kg: Kilogram
OAD: Oral antidiabetic drug
T1DM: Type 1 diabetes mellitus
